# Antibacterial properties of polydopamine-modified ZnO nanoparticles composite films for oral therapeutic applications

**DOI:** 10.1186/s11671-025-04421-5

**Published:** 2026-01-06

**Authors:** Lei Jin, Junping Fan, Xin Zhu, Shen Wang, Yaoxi Yang, Dongfang Li

**Affiliations:** 1 School of Stomatology, Jiangxi Medical College, Nanchang University, Nanchang, 330006 People’s Republic of China; 2 Jiangxi Provincial Key Laboratory of Oral Diseases, Nanchang, 330006 People’s Republic of China; 3 Jiangxi Provincial Clinical Research Center for Oral Diseases, Nanchang, 330006 People’s Republic of China; 4 Jinan Stomatology Hospital, 101 Jingliu Road, Shizhong District, Jinan City, 250000 Shandong Province People’s Republic of China; 5 Affiliated Hospital of Jinggangshan University, Ji’an, 343000 People’s Republic of China

**Keywords:** Corn prolamin, Zinc oxide nanoparticles, Polydopamine, Drug release, Antibacterial activity

## Abstract

In this work, a solvent evaporation casting approach was used to prepare ZnO@PDA/Zein nanocomposite films, where polydopamine-modified zinc oxide nanoparticles (ZnO@PDA NPs) were incorporated into a zein matrix as the film-forming agent. This nanocomposite film adhered uniformly to the surface of oral therapeutic instruments and exhibited good antibacterial effects. At a doping concentration of 1.2 mg/mL, the film exhibited an antibacterial effect against Gram-negative bacteria such as *Escherichia coli (E. coli)*, and at a doping concentration of 0.6 mg/mL, the film exhibited antibacterial effects against Gram-positive bacteria such as *Staphylococcus aureus (S. aureus)* and *Streptococcus mutans (S. mutans)*, along with good biocompatibility. This study introduces a novel ZnO@PDA/Zein nanocomposite film with enhanced antibacterial efficacy and biocompatibility, offering a promising solution for preventing biofilm formation on oral therapeutic instruments.

**Supplementary Information:**

The online version contains supplementary material available at 10.1186/s11671-025-04421-5.

## Introduction

The oral cavity is a complex and dynamic environment in which oral biofilms form and adhere to the surfaces of teeth and dental materials. Oral biofilms are involved in the pathogenesis of caries, periodontitis, secondary caries and peri-implantitis, which can lead to failure of clinical treatment [1]. Currently, the main clinical methods for controlling dental plaque biofilms are mechanical debridement (scaling and sandblasting) and adjunctive therapy (chlorhexidine) [2, 3]. While the aforementioned methods have proven therapeutic, reaching the narrow parts of treatment devices poses a challenge, such as orthodontic brackets and implants for mechanical removal [4]. In addition, the use of antibacterial agents may cause bacterial resistance [5]. Notably, smooth teeth and restored surfaces have self-cleaning effects.

However, when treatment devices, including orthodontic brackets, cyst plugs, and implant healing abutments, remain in a patient’s mouth for a long period, oral microorganisms easily adhere to surfaces and aggregate [6–8]; this leads to an imbalance in the oral flora and subsequent adverse reactions, such as partial demineralization of dental hard tissues, peri-implantitis, and delayed bone healing [9, 10]. Notably, preventing oral biofilm infection rather than treating it is particularly important [11]. Therefore, controlling microbial infection and inhibiting the proliferation of pathogenic bacteria are major challenges in the clinical diagnosis and treatment of oral diseases [12].

Nanotechnology is a research hotspot in modern materials science with a variety of novel applications, from chemical product design, food processing, and agricultural production to complex medical and health applications [13]. The term nanoparticles (NPs) refers to particles with a diameter of 1–100 nm [14]. As nanoscience and technology progress, metal-based nanostructured materials, such as copper NPs, silver NPs and ZnO NPs, have frequently been employed in oral antibacterial treatment [15].

ZnO NPs have become among the most promising antibacterial materials because of their broad-spectrum antibacterial efficacy, prolonged stability, and excellent biocompatibility, although their toxicity is concentration dependent [16]. ZnO NPs have potent antibacterial properties against both Gram-positive and Gram-negative bacteria, and the unique antibacterial mechanism of ZnO NPs, including the release of Zn^2+^ and the generation of reactive oxygen species (ROS), makes the development of resistance difficult, which is highly important for preventing bacteria from evolving into super bacteria [17].

However, ZnO NPs are typically physically blended within a polymer matrix, leading to inadequate dispersion and an increased tendency for leaching. These factors adversely affect the overall properties of the resulting composites [18]. Surface modification is an effective approach to enhance the interaction between NPs and polymers, thus increasing their dispersion stability [19–21].

Polydopamine (PDA), a synthetic melanin polymer generated by self-polymerization of a monomer under weakly alkaline conditions, is biocompatible and the main pigment of natural melanin [22]. Furthermore, PDA has extraordinary adhesion ability and can securely bond to the surfaces of metallic nanomaterials, yielding a novel type of functional metal-based polydopamine (MPDA) nanomaterial that has promising functions and wide application prospects [23]. Owing to the complexity of the oral environment, most antibacterial drugs cannot be retained for a long period to exert their antibacterial effects [18]. Moreover, ZnO NPs are prone to agglomeration; thus, a PDA surface coating approach can considerably enhance their dispersion within the polymer matrix [24, 25]. More importantly, PDA is a natural reductant that protects against ZnO NP-induced oxidative damage [26]. Therefore, the use of these materials to fabricate a new type of antibacterial film that can be attached to the surface of oral treatment instruments is feasible.

Zein, a natural protein derived from corn, has garnered increasing interest for the development of drug delivery systems and medical device coatings [27, 28]. Compared with other protein-based NPs, zein has unique properties, such as reproducible production, biodegradability, biocompatibility, and the ability to immobilize drugs and hydrophobic and hydrophilic ligands on its surface [29–31]. However, pure zein cannot achieve satisfactory antibacterial effects [32, 33]. Additionally, zein-based systems are often initially impacted by the rapid burst release of the active ingredients from certain single-phase nanocomposites [34]. Zein can leverage its excellent ability to self-assemble to incorporate ZnO@PDA NPs into nanocomposite films, thus endowing pure zein films with antibacterial properties [35]. Zein can additionally be employed in local tissue drug delivery systems (DDSs) to participate in drug release by exploiting its ability to stabilize bioactive compounds [34, 36].

In this study, we synthesized ZnO@PDA/Zein nanocomposite films, the main components of which are PDA, ZnO NPs, and Zein. Zein served as the primary film-forming and stabilizing agent, ZnO NPs exerted antibacterial effects, and PDA reduced the cytotoxicity of the ZnO NPs. A series of techniques were used to study their surface characteristics; chemical composition; and degradation, adhesion, and ion release properties. Moreover, we evaluated the antibacterial properties and biocompatibility of the nanocomposite film to demonstrate its feasibility for use as a dental material for antibacterial surface treatment.

## Experimental section

### Materials

In this study, ZnO NPs (Ningbo, China), corn alcohol-soluble protein powder (Zein, Nanjing, China), anhydrous ethanol (Shanghai Aladdin Biochemical Technology Co., Ltd., China), dopamine (DA; Sinopharm Group Chemical Reagent Co., Ltd., China), *Staphylococcus aureus* (*S. aureus)* dried powder (BNCC186335, BeNa Culture Collection Co., Ltd., China), *Escherichia coli (E. coli)* dried powder (BNCC310011, BeNa Culture Collection Co., Ltd., China), *Streptococcus mutans (S. mutans)* dried powder (BNCC336931, BeNa Culture Collection Co., Ltd., China), Luria–Bertani (LB) broth (Beckman Biotechnology Co., Ltd., China), Columbia blood agar (Beckman Biotechnology Co., Ltd., China), and LB liquid medium (Beijing Biotechnology Co., Ltd., China) were used.

### Preparation of the ZnO@PDA/Zein nanocomposite film

#### Preparation of ZnO@PDA NPs

First, 10 mg of ZnO NPs were dispersed in Tris (hydroxymethyl) aminomethane buffer (50 mM, pH 8.5, 50 mL) and stirred with ultrasonication for 10 min to obtain a ZnO NPs suspension [37]. Additionally, 5 mg of DA was added. The mixture was stirred at 300 rpm for 16 h. After the reaction, the ZnO@PDA NPs were collected via centrifugation at 8000 rpm for 10 min at 25 °C. The resulting pellet was washed three times with deionized water. The washed NPs were then collected and subjected to freeze-drying.

#### Preparation of the Zein film-forming solution

Three quantities of zein powder (500 mg, 750 mg, and 100 mg) were separately dissolved in 20 mL of 80% (v/v) aqueous ethanol with stirring until completely dissolved [38]. Each solution was then concentrated to a volume of 10 mL by evaporating the solvent at 50 °C and 600 rpm, yielding zein film-forming solutions at concentrations of 50, 75, and 100 mg/mL. These solutions were preserved at 4 °C for subsequent utilization.

#### Preparation of the ZnO@PDA/Zein nanocomposite film

**Fig. 1:**
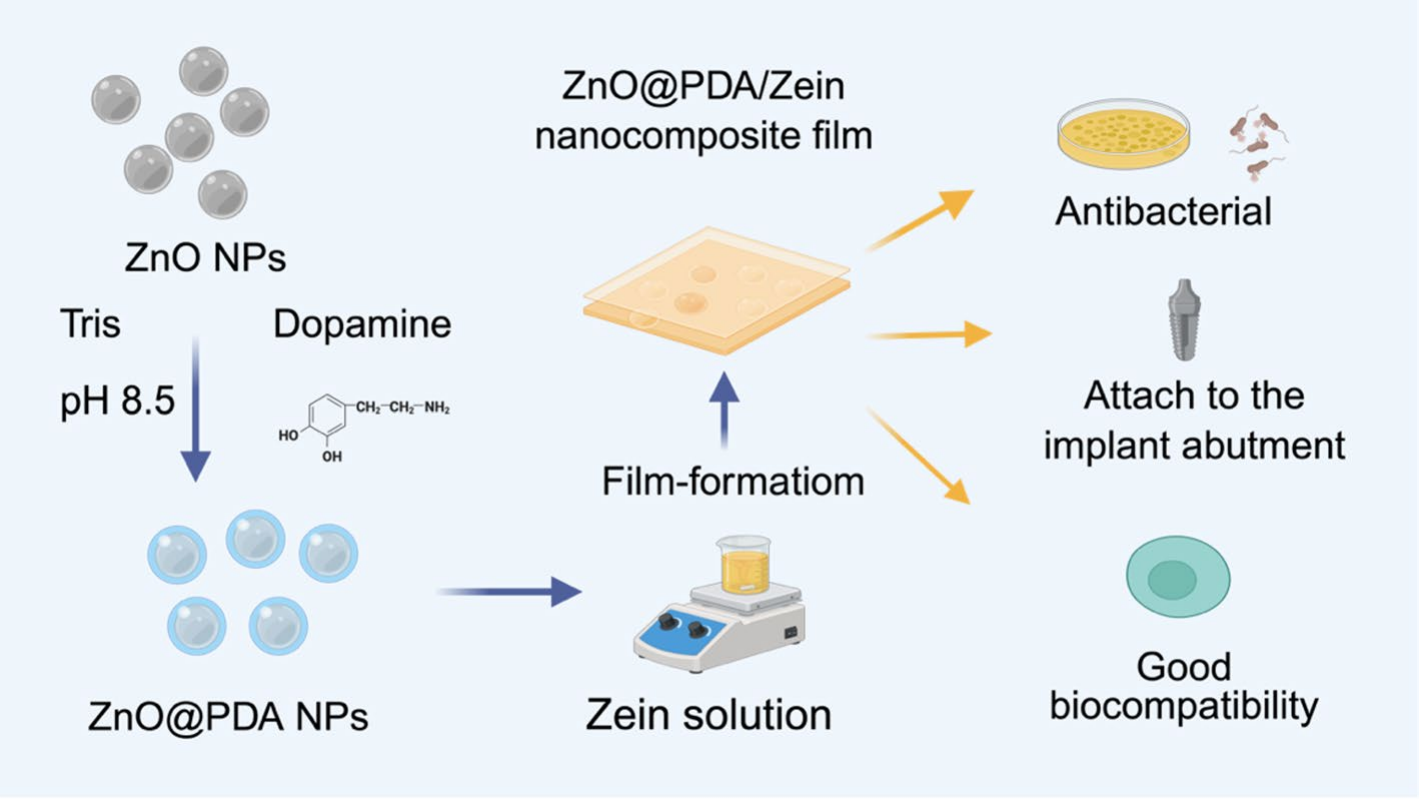
Scheme depicting the synthesis and biological applications of the ZnO@PDA/Zein nanocomposite film (The figure was created using BioRender.com. The publication lists are provided in supplemental file 1.)

The ZnO@PDA/Zein nanocomposite film was prepared using the solution casting technique [34]. A total of 6 mg of ZnO@PDA NP powder was incorporated into 10 mL of 50 mg/mL zein film-forming solution. The mixture was then stirred at 600 rpm using a magnetic stirrer for 10 min at ambient temperature to ensure thorough mixing. Subsequently, 1 mL of the solution intended for film formation was evenly applied to the implant healing bases via a rubber-tipped dropper. The samples were then placed in a temperature-controlled chamber maintained at 25 °C to observe the film-forming process directly (Fig. 1).

### Characterization

#### X-ray diffraction (XRD) analysis

The XRD patterns of the nanocomposite film samples were acquired using an X-ray diffractometer (D8ADVANCE, Bruker, Germany) fitted with a Cu target ceramic X-ray tube. The accelerating voltage was 40 kV, the current was 40 mA, and the scanning range was from 10° to 80° with a step size of 0.02°.

#### **Fourier transform infrared** (**FTIR) analysis**

The FTIR spectra of the nanocomposite film samples were recorded using a FTIR spectrometer (Nicolet iS50, Thermo Fisher, USA). The scanning parameters included a scanning range of 4000–400 cm^− 1^, 64 scans, and a resolution of 4 cm^− 1^. The spectral data were collected from each sample three times under identical conditions.

#### Transmission electron microscopy (TEM) analysis

The powdered sample was dispersed in deionized water via ultrasonication, followed by vortex mixing to obtain a homogeneous suspension. An aliquot of the suspension was then drop-cast onto a copper grid and allowed to dry at room temperature before TEM observation (JEM-F200, JEOL, Japan).

#### Scanning electron microscopy (SEM) analysis

SEM (Sigma 560, Carl Zeiss Microscopy, Germany) analysis was conducted to observe the microstructures of the nanocomposite film samples. Prior to capturing images, the nanocomposite film was affixed to an aluminium plate and subsequently covered with a slender layer of gold coating under a vacuum environment to render it conductive.

#### Water contact angle measurements

The water contact angle of the ZnO@PDA/Zein nanocomposite film was quantified via the drop method using a water contact angle-measuring device (DSA100, KRUSS, Germany). Three different positions on the surface of each sample were randomly selected, and a small amount of distilled water was introduced at each position with a microsyringe such that the water contacted the surface of the sample. After a 5-second pause, the water droplet on the sample surface was observed under a microscope and photographed to record the real-time left and right side water contact angles within a 10 s window. The average water contact angle was then calculated.

#### Degradation of the ZnO@PDA/Zein nanocomposite film

Three millilitres of film-forming solution (containing 50 mg/mL zein, 1.25 mg/mL ZnO@PDA NPs, and 80% ethanol) were placed into a cast and dried for 24 h to form a nanocomposite film. The weight was subsequently recorded as W_1_. The nanocomposite film was subsequently placed in artificial saliva (pH 7.1) and soaked for 24 ± 1 h. After soaking, the nanocomposite film was placed back in a 25 °C constant temperature box, dried at a constant temperature for 24 h, and weighed, and the weight was recorded as W_2_. All the tests were repeated three times (1).

1 $$ {\text{Mass}}\;{\text{loss}}\;{\text{rate}}\;(\% ) = \left( {{\text{(W}}_{{\text{2}}} - {\text{W}}_{{\text{1}}} {\text{)/W}}_{{\text{1}}} } \right) \times {\text{100}} $$

#### Zn^2+^ release

Two millilitres of film-forming solution (composed of 50 mg/mL zein, 5 mg/mL ZnO@PDA, and 80% ethanol) were placed into a cast and dried to form a nanocomposite film, which was immersed in 50 mL of aqueous solution. After 4 h, 12 h, 24 h, 48 h, 72 h, 5 days, 7 days, and 14 days, 2 mL of the aqueous solution was removed and diluted 5-fold, and the Zn^2+^ concentration in the diluted solution was determined via an inductively coupled plasma mass spectrometer instrument. The overall release rate of Zn^2+^ was determined via the following formula (2):

2 $$ {\text{Zn}}^{{{\text{2 + }}}} \;{\text{release}}\;{\text{rate}}\;(\% ){\text{ = }}\left( {\sum\limits_{1}^{{{\text{n}} - 1}} {{\text{10C}}_{{{\text{(n}} - {\text{1)}}}} } {\text{ + 5}}\left( {{\text{V}}_{{\text{0}}} - {\text{2(n}} - {\text{1)}}} \right){\text{/M}}_{{\text{i}}} } \right) \times 100 $$

where M_i_ represents the initial content of ZnO@PDA NPs in the sample; n denotes the number of measurements; V_0_ signifies the cumulative volume of the release medium (50 mL); and Cn indicates the Zn^2+^ concentration in the nth measurement.

### Adhesion and mechanical properties of the ZnO@PDA/Zein nanocomposite film

Two millilitres (2 mL) of ZnO@PDA NPs solution, 50 mg of 50 mg/mL zein solution, 100 mg of 100 mg/mL zein solution, 0.6 mg of 0.6 mg/mL ZnO@PDA NPs solution, and a solution of ZnO@PDA/Zein nanocomposite film (composed of 50 mg/mL zein and 0.6 mg/mL ZnO@PDA NPs) were dropped onto titanium plates separately, and implant healing abutments were used to determine the film formation time. The titanium plates loaded with the nanocomposite films were subsequently positioned onto a nanoscratch tester (Anton Paar UNHT, Austria), and scratch tests were conducted at a speed of 2 mm/min. All the experiments were conducted in triplicate for consistency.

### Antibacterial properties of the ZnO@PDA/Zein nanocomposite film

The lyophilized bacterial powder was added to the activation solution and incubated at 37 °C for 10 min with constant shaking. Then, 100 µL of the bacterial suspension was inoculated onto the agar medium surface and incubated at 37 °C for 16 h under aerobic conditions. A single colony was subsequently scraped from the surface of the agar medium via an inoculation loop and resuspended in 5 mL of LB broth. After thorough mixing, 100 µL of the bacterial suspension was transferred to 4.9 mL of fresh LB broth medium. This mixture was then shaken at a constant temperature of 37 °C and a speed of 200 rpm in a shaker for 16 h. The optical density at 600 nm (OD 600 nm) was measured via a microplate reader, and the bacterial suspension was diluted to a concentration of 10^7^ CFU/mL for subsequent use.

Antibacterial activity test: ZnO@PDA/Zein nanocomposite films containing different concentrations of ZnO@PDA NPs were added to separate wells of a 96-well plate. To each well, 20 µL of fresh bacterial suspension was added, and the plate was incubated at a constant temperature of 37 °C for 2 h. Subsequently, 1 mL of sterile PBS was added to each well to resuspend the surviving bacteria. Then, 100 µL of the resuspended *E. coli* and *S. aureus* bacterial suspensions were evenly spread on the surface of the agar medium, and 100 µL of the resuspended *S. mutans* bacterial suspension was evenly spread on the surface of Columbia blood agar medium. After incubation at a constant temperature of 37 °C in a bacterial incubator for 16 h, the numbers of colonies of *E. coli*,* S. aureus*, and *S. mutans* on the media were counted using the colony counting method. The antibacterial activity of different samples was compared, with each experiment repeated three times.

3 $${\text{Microbial}}\;{\text{reduction}}\;{\text{percentage}}\;(\% ) = \left( {({\text{Control}}\;{\text{CFU}} - {\text{Test}}\;{\text{CFU}})/{\text{Control}}\;{\text{CFU}}} \right) \times 100 $$

where the control CFU represents the number of CFUs incubated with PBS and where test CFU denotes the number of CFUs at ZnO@PDA NPs concentrations of 0, 0.6, 1.2, or 2.4 mg/mL.

### Biocompatibility of the ZnO@PDA/Zein nanocomposite film

Mouse embryo osteoblast precursor cells (MC3T3-E1 cells) were added to 5 mL of α-MEM containing 10% fresh foetal bovine serum (FBS) and cultured for 24 h under 5% CO_2_ at 37 °C. The experimental groups included 0.6 mg/mL ZnO@PDA NPs, 0.6 mg/mL ZnO NPs, 1.2 mg/mL ZnO@PDA NPs, and 1.2 mg/mL ZnO NPs, whereas the control group was 5% pure zein, and the blank control group was a medium and CCK-8 solution only. Each group received 2 mL of cell culture medium.

After 24 h, the 24-well plate was removed, and the original medium inside the plate was removed. The plate was washed once with PBS, and 2 mL of fresh α-MEM containing 10% FBS and 20 µL of CCK-8 solution were added to each well. The plate was then incubated in a constant-temperature CO_2_ incubator for another 2 h. The supernatant was subsequently added to a new 24-well plate, and the absorbance (optical density; OD) at 450 nm was measured with a microplate reader. A live/dead double-staining kit was used for staining analysis of the experimental groups, and the stained cells were examined and documented using a confocal laser scanning microscope. The above experiment was independently repeated three times.

4 $${\text{Cell}}\;{\text{viability}}\;{\text{rate}}\;(\% ) = \left( {({\text{Sample}}\;{\text{OD}} - {\text{Blank}}\;{\text{OD}})/({\text{Control}}\;{\text{OD}} - {\text{Blank}}\;{\text{OD}})} \right) \times 100 .$$

where the sample OD refers to the OD value of the experimental group, the blank OD denotes the OD value of the blank control group, and the control OD represents the OD value of the control group.

### Statistical analysis

Graphical plotting was performed using Origin 2024 software, and data analysis was conducted using SPSS 26.0 (IBM, Armonk, NY, USA). All experiments were repeated three times. The measurement data are expressed as the means ± standard deviations (means ± SDs). For comparisons between two groups, a one-sample t test was used. For comparisons among multiple groups, one-way analysis of variance (one-way ANOVA) was employed. Before performing the analysis of variance, Levene’s test was used to verify the homogeneity of variances. If the condition of variance homogeneity was satisfied, an F test was used for intergroup comparisons; if not, Welch’s test was adopted. The analysis of variance results revealed significant differences among the groups. Tukey’s post hoc test was further used for pairwise comparisons. All tests were two-tailed, and the significance level was set at 0.05. A p value < 0.05 was considered to indicate a significant difference, with *p < 0.05, **p < 0.01, and ***p < 0.001.

## Results

### Characterization

#### XRD

**Fig. 2:**
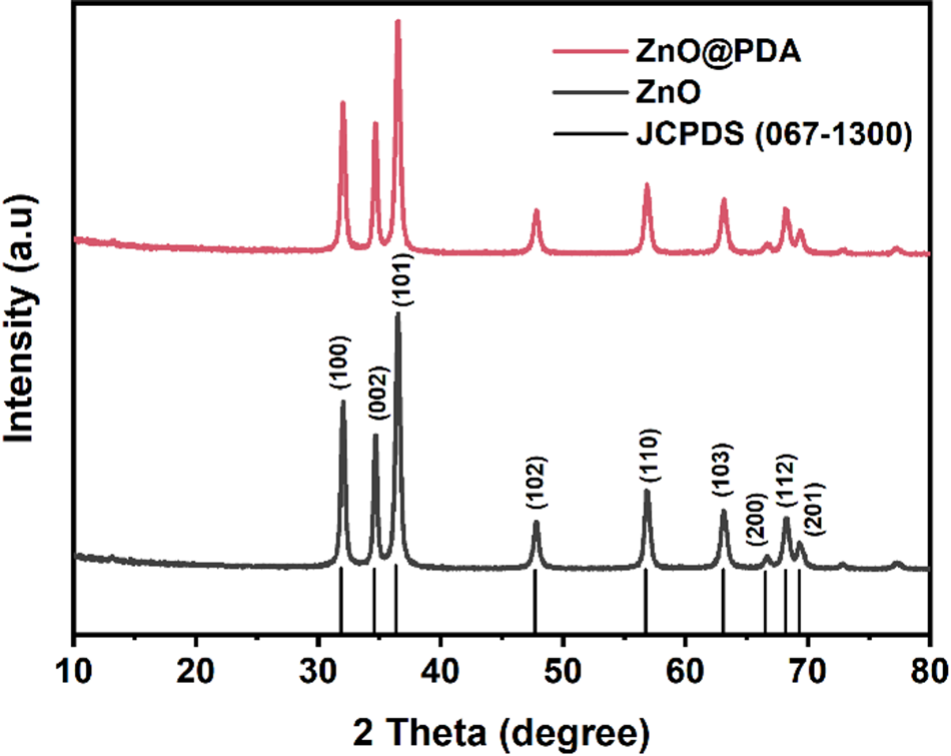
XRD patterns of the ZnO NPs, ZnO@PDA NPs, Zein, and ZnO@PDA/Zein nanocomposite films

The XRD spectrum of the ZnO NPs (Fig. 2) showed peaks at 31.84°, 34.55°, 36.35°, 47.69°, 56.74°, 63.09°, 66.56°, 68.16°, and 69.28°, corresponding to the (100), (002), (101), (102), (110), (103), (200), (112), and (201) planes of the ZnO NPs, respectively. These diffraction peaks confirmed the hexagonal wurtzite structure (JCPDS 067-1300) of the ZnO NPs and the presence of crystalline ZnO NPs within the ZnO@PDA NPs. The XRD pattern of the ZnO NPs showed no additional peaks, suggesting that the crystalline ZnO NPs were free from impurities [39]. The XRD pattern of the ZnO@PDA NPs had peaks similar to those of the ZnO NPs, although the intensities of these peaks notably decreased [40]. This reduction in intensity was due to the self-polymerization of DA to form an amorphous PDA coating on the surface of the ZnO NPs, which weakened the intensities of the substrate diffraction peaks [41]. However, the shapes and angles of the diffraction peaks remained largely unchanged in the ZnO@PDA NPs sample, suggesting that PDA was deposited on the ZnO NPs surface but did not alter its overall structure.

#### FTIR

**Fig. 3:**
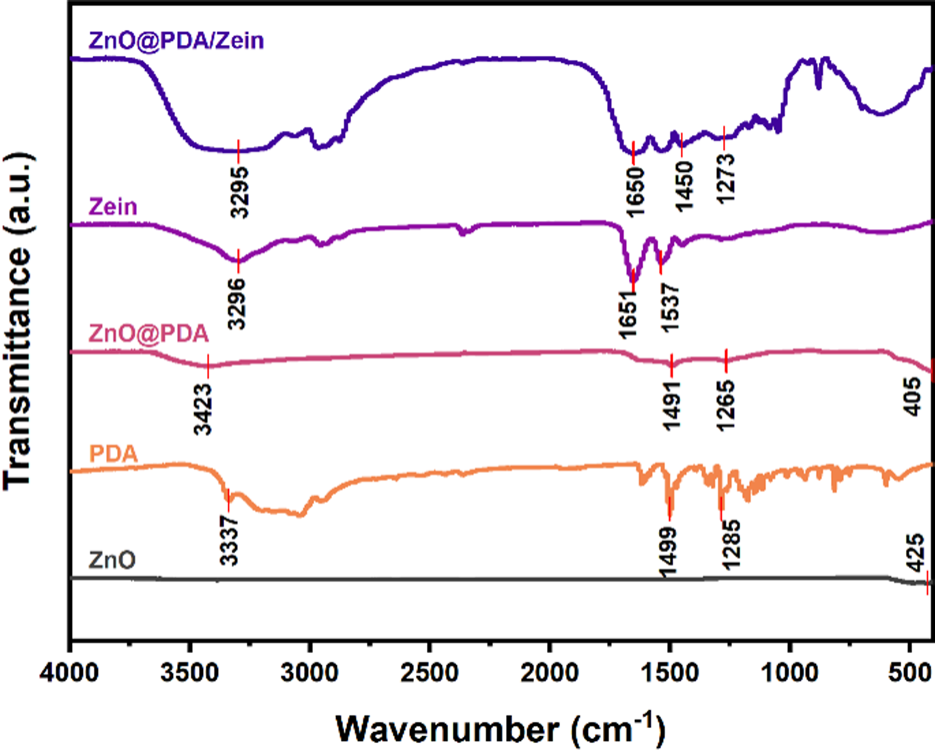
FTIR spectra of the ZnO NPs, ZnO@PDA NPs, Zein, and ZnO@PDA/Zein nanocomposite film

FTIR was used to examine the structural characteristics of zein, ZnO@PDA/Zein NPs, ZnO@PDA NPs, and pure ZnO NPs. Figure 3 shows the spectrum of the ZnO NPs, which features an absorption peak near 425 cm^− 1^ attributed to the stretching vibration of the Zn–O bond. The peaks at 1285, 1499, and 3337 cm^− 1^ correspond to the carbon–oxygen single bond (C–O), the carbon‒carbon double bond (C=C) within the aromatic ring of PDA, and the stretching vibrations of the hydroxyl (–OH) or amino groups (–NH) of PDA, respectively. Additionally, the characteristic absorption peaks of the ZnO@PDA NPs were evident near 1265, 1491, and 3423 cm^− 1^, corresponding to C–O, C=C, and –OH/–NH, respectively [24]. These results verify the successful preparation of the ZnO@PDA NPs. Compared with those of ZnO NPs and PDA, the peaks of ZnO@PDA NPs were similar to those of PDA, which was attributed to the presence of the PDA shell [42]. The zein molecule displayed typical protein absorption peaks in the amide A (3296 cm^− 1^), amide I (1651 cm^− 1^), and amide II bands (1537 cm^− 1^), corresponding to the stretching vibrations of the carbonyl (C=O), –NH, and cyano (–CN) groups, respectively. The peak from 3500 to 2800 cm^− 1^ corresponds to the –OH or –NH groups of PDA and zein, respectively, whereas the peaks at 1650 and 1450 cm^− 1^ are likely attributed to the amide I and amide II bands of zein, respectively [43]. Additionally, the peak at 1273 cm^− 1^ corresponds to the C–O stretching vibration of PDA. These data confirmed the successful formation of the ZnO@PDA/Zein nanocomposite film. In contrast to zein, the ZnO@PDA/Zein nanocomposite film presented peaks at 3295 cm^− 1^ for the amide A band, 1650 cm^− 1^ for the amide I band, and 1450 cm^− 1^ for the amide II band, along with changes in both the peak values and configurations [44]. This could be due to the formation of noncovalent hydrogen bonds between the PDA and zein molecules [45]. The influence of these hydrogen bonds amplifies the stretching vibration of –OH, resulting in a shift towards lower wavenumbers [46]. Importantly, more hydrogen bonds can enhance ZnO@PDA/Zein NPs encapsulation, thus increasing the stability of the nanocomposite film [47].

#### TEM

**Fig. 4:**
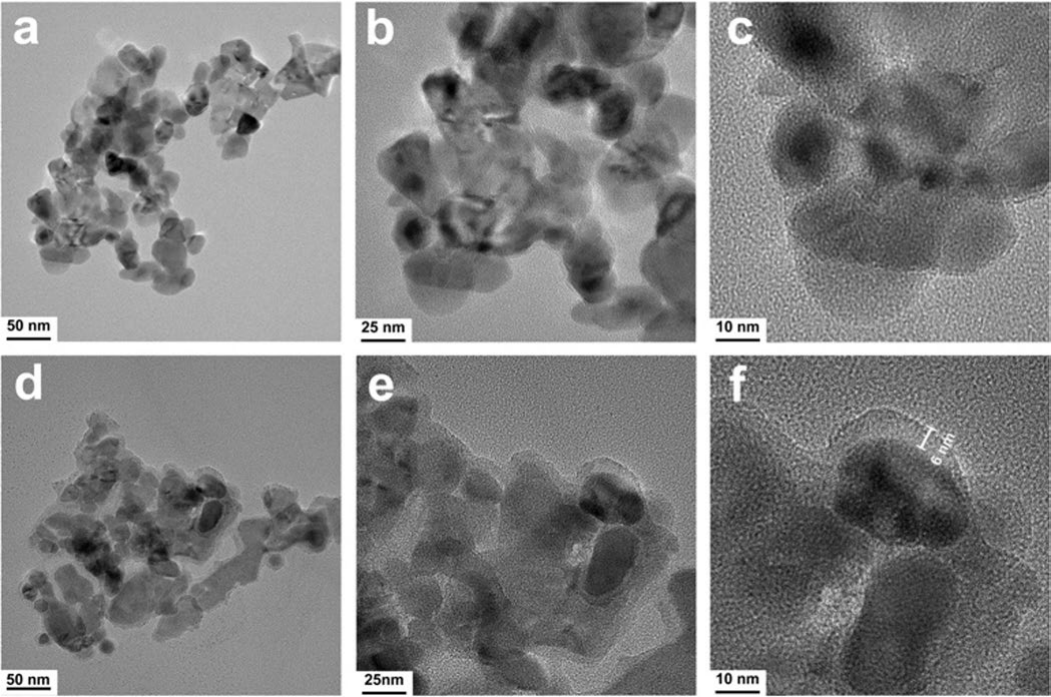
TEM images of ZnO NPs (**a**, **b**, **c**) and ZnO@PDA NPs (**d**, **e**, **f**)

The morphological characteristics of the ZnO NPs and ZnO@PDA NPs were observed via TEM. As depicted in Fig. 4a–c, the ZnO NPs exhibit a rounded crystalline morphology with an average diameter of approximately 20 nm, which aligns with previous findings [48, 49]. The morphology of the ZnO@PDA NPs was not considerably different, and these particles have a diameter of approximately 26 nm. Figure 4f shows that a 6 nm thick PDA layer was deposited on the ZnO NP surface. Thus, the PDA layer resembles a shell encapsulating ZnO NPs. In Fig. 4c, the surface of the pure ZnO NPs appears smooth, and the edge image of the ZnO NPs is sharp. In contrast, Fig. 4d shows that the edge image of the ZnO NPs is notably rough, which is a result of PDA deposition. The TEM images effectively showed that the ZnO NPs were modified with PDA. Research has indicated that PDA can be deposited on nearly all material surfaces because of the easy polymerization of DA into PDA through π-bonding, hydrogen bonding, and covalent bonding interactions under alkaline conditions [50].

#### SEM

**Fig. 5:**
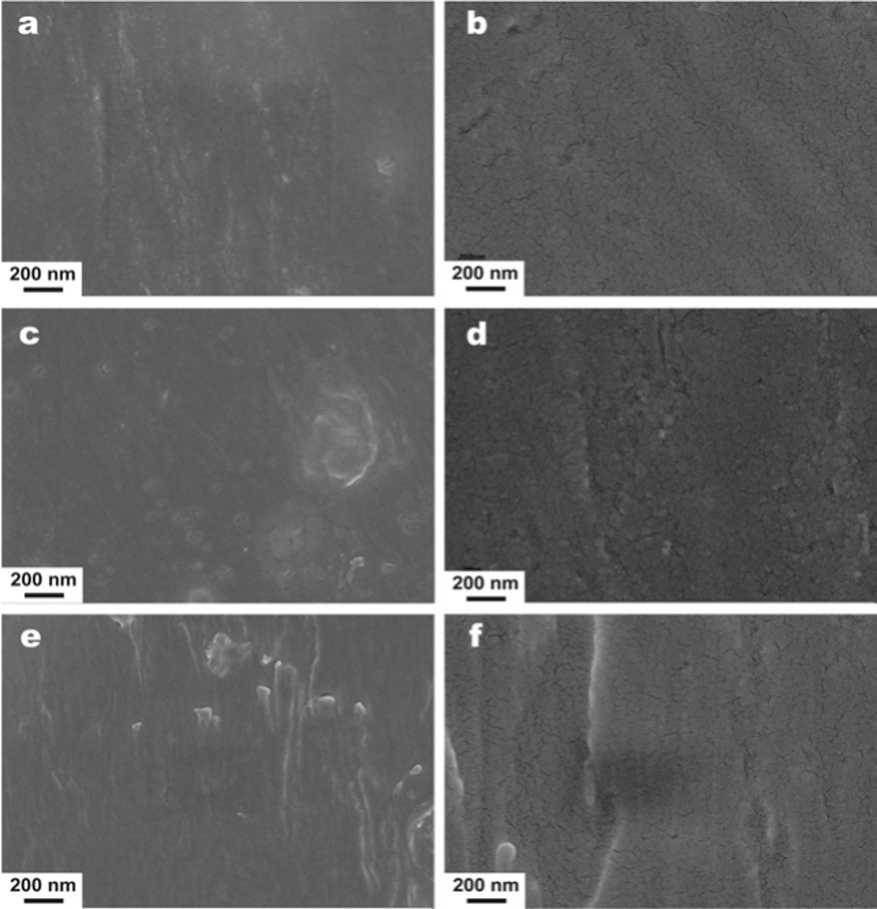
SEM images of the nanocomposite films containing 0.5 mg/mL ZnO@PDA NPs (**a**, **b**), 1.25 mg/mL ZnO@PDA NPs (**c**, **d**) and 2.5 mg/mL ZnO@PDA NPs (**e**, **f**)

SEM analysis revealed the surface morphology of 50 mg/mL zein in 80% ethanol and the nanocomposite films with ZnO@PDA NPs concentrations of 0.5, 1.25, and 2.5 mg/mL. As depicted in Fig. 5a and b, the composite film with 0.5 mg/mL ZnO@PDA NPs had a dense and smooth surface structure. A pure zein film forms during cast film drying. Because the rate of ethanol evaporation from the dispersion is faster than that of water, the water content and hydrophilicity of the solvent gradually increase. These alterations, coupled with an increase in protein concentration, enhance the hydrophobic interactions among zein molecules, prompting them to aggregate and self-assemble into thin films [51]. Figure 5c, d show the surface morphology of the nanocomposite film containing 1.25 mg/mL ZnO@PDA NPs. The images revealed a uniform distribution of ZnO@PDA NPs throughout the nanocomposite film matrix. Overall, the electrostatic repulsion between the NPs and protein molecules allows the ZnO@PDA NPs to be uniformly distributed within the nanocomposite film (Fig. 5e, f), illustrating the surface morphology of the nanocomposite film containing 2.5 mg/mL ZnO@PDA NPs, where the grooves of the nanocomposite film are deeper and wider; this is likely due to the larger quantity of ZnO@PDA NPs aggregating with zein protein molecules. The high concentration of ZnO@PDA NPs results in their clustering during the drying process to form a film, and the casting process further accelerates this phenomenon.

#### Water contact angles

**Fig. 6:**
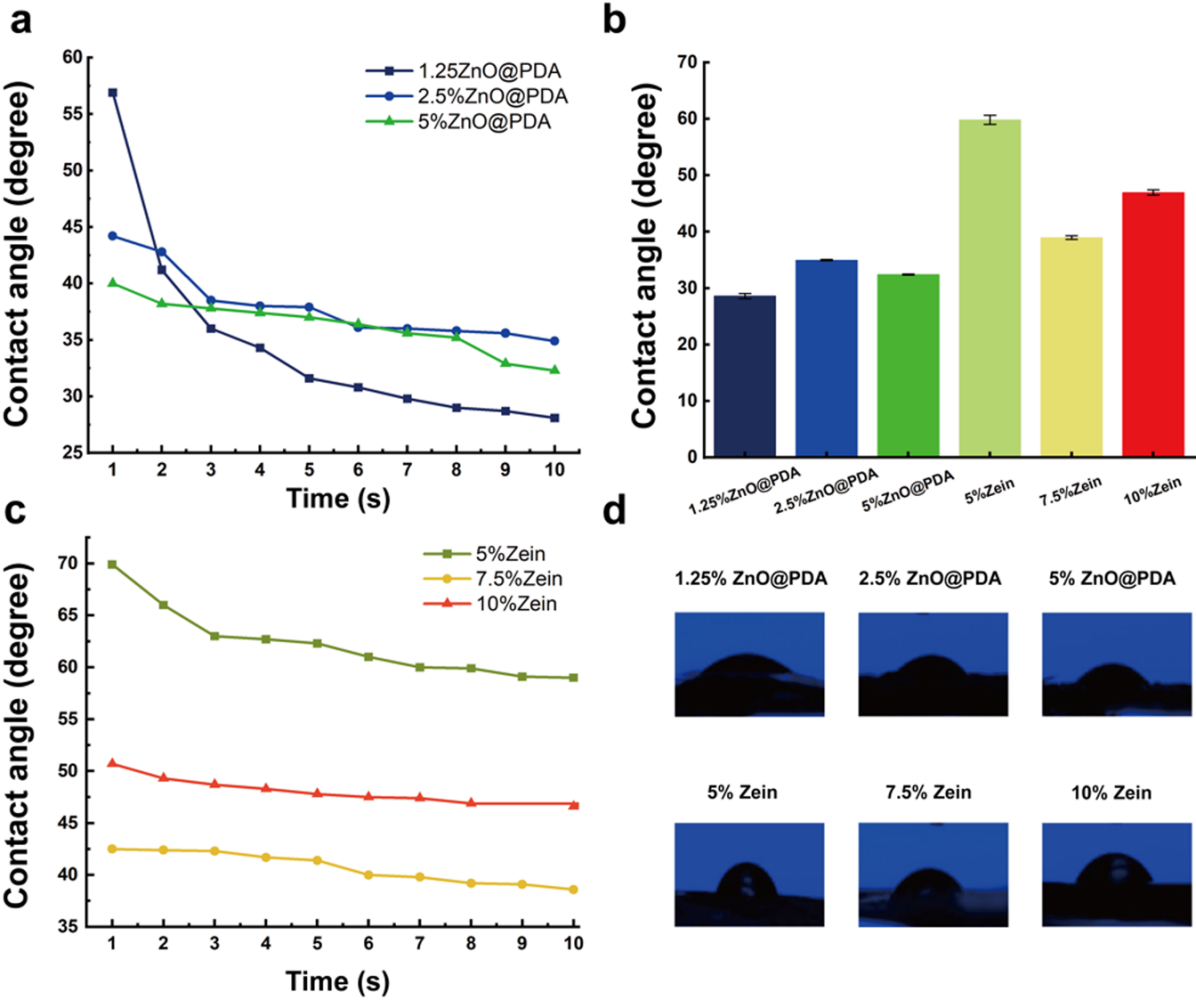
**a** Changes in the water contact angle of the ZnO@PDA/Zein nanocomposite film over 10 s. **b** Water contact angles of ZnO@PDA NPs and zein at different concentrations. **c** Changes in the water contact angle of the pure zein film. **d** Images of the water contact angles

Figure 6a shows the ZnO@PDA/Zein nanocomposite film prepared with ZnO@PDA NPs contents of 1.25%, 2.5%, and 5% mg/mL in 80% (v/v) ethanol and 50 mg/mL zein. Additionally, Fig. 6c shows the average water contact angles of pure zein films with concentrations of 5%, 7.5%, and 10% in 80% (v/v) ethanol. The average water contact angle of the 5% pure zein film was 69°, but as the concentration of zein increased to 10%, the average water contact angle decreased to 50°. This is because although zein molecules contain many hydrophobic moieties (such as glutamine residues) [52], as the concentration of zein increases, the water contact angle decreases [38]. However, during the solvent evaporation process, owing to the interactions among and self-aggregation of the hydrophobic moieties in the zein molecules (such as proline, leucine, isoleucine, and alanine residues), the hydrophilic groups are forced to face outwards. With increasing zein concentration, the number of hydrophilic groups exposed on the surface increases, resulting in an increase in the hydrophilicity and a decrease in the hydrophobicity of the pure zein film [53].

To ensure that the nanocomposite film was not rapidly degraded in the mouth, a zein concentration of 5% was chosen for subsequent experiments. When 1.25 mg/mL ZnO@PDA NPs were added to the nanocomposite film, the water contact angle was 56.9°, which decreased to 31.3° after 5 s. When 2.5 mg/mL ZnO@PDA NPs were added, the water contact angle was initially 44.2° but decreased to 37.9° after 5 s. The addition of ZnO@PDA NPs to the pure zein film reduced the surface water contact angle, possibly for two reasons. First, both PDA and ZnO NPs are hydrophilic substances that easily attract water molecules [54, 55]. Few surface –OH groups of the ZnO NPs are modified by PDA, and phenolic hydroxyl, amino, and other hydrophilic groups are present [56]. Thus, the ZnO@PDA/Zein nanocomposite film maintains a high degree of hydrophilicity. Second, the ZnO NPs have photocatalytic properties and can easily generate a pair of electron holes under photocatalytic action. The reaction between the holes and bridging oxygen ions results in the formation of oxygen vacancies [57]. Water molecules in the air then dissociatively adsorb to the oxygen vacancies, becoming chemically hydrophilic, absorbing more moisture from the air, and causing the water contact angle of the film to decrease. Figure 6b shows that the water contact angle of the ZnO@PDA/Zein nanocomposite film decreased within 5 s, which is attributed to the presence of a high concentration of ZnO@PDA NPs, which resulted in the surface of the nanocomposite film becoming rough and the surface porosity increasing.

#### **Degradation of the ZnO@PDA/Zein nanocomposite film**

**Fig. 7:**
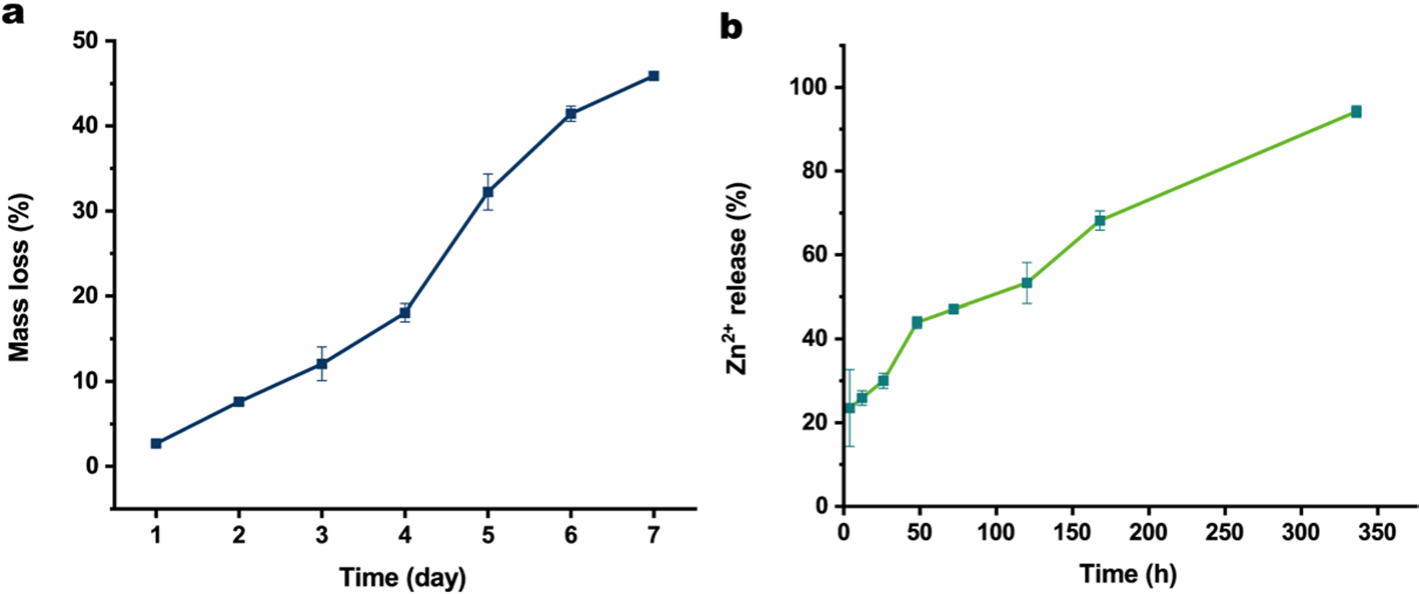
**a** Mass loss of the ZnO@PDA/Zein nanocomposite film. **b** Degradation of the nanocomposite film over 7 days and the release of Zn^2+^ from the nanocomposite film over 14 days

An ideal nanocomposite film with long-term antibacterial effects on the surface of oral instruments needs to remain stable within the complex oral environment while also self-degrading to a certain degree. In this study, the degradation of the nanocomposite film was simulated in artificial saliva in vitro over 7 days, as shown in Fig. 7a. After drying, moistening, and redrying in artificial saliva for 7 days, the mass loss rate of the nanocomposite film was 55%. This demonstrates that the ZnO@PDA/Zein nanocomposite film has good stability and self-degradation properties in saliva.

#### Zn^2+^ release

During the evaluation of the Zn^2+^ release capacity of the ZnO@PDA/Zein nanocomposite film, as shown in Fig. 7b, the film released approximately 23% of the Zn^2+^ within a 4-hour period. During the acute release phase, the average release rate was 5% per hour. In contrast, the period from 48 to 72 h was characterized by relatively slow release, with an average hourly release rate of approximately 0.13%. From 72 h to 14 days, the release rate subsequently further decreased. The average release rate was 0.11% per hour. By the end of this stage, the cumulative amount of Zn^2+^ released had reached 94%. One possible reason for this is that organic matter has high hydrophilicity and a relatively small molecular size, resulting in a relatively fast release rate [58].

### Adhesion and mechanical properties of the ZnO@PDA/Zein nanocomposite film

**Fig. 8:**
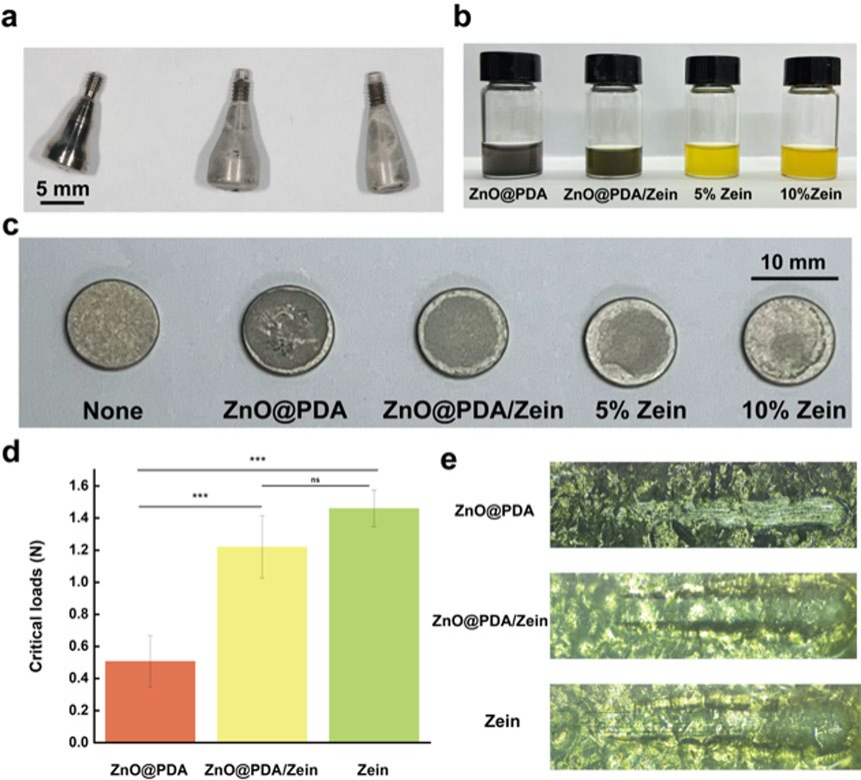
**a** ZnO@PDA/Zein nanocomposite film attached to the implant healing abutment. **b** ZnO@PDA/Zein nanocomposite film in a liquid state. **c** ZnO@PDA/Zein nanocomposite film attached to a titanium plate. **d** Critical binding forces at which the ZnO@PDA/Zein nanocomposite film fractured during the scratch test. **e** Images of the scratches under a light microscope; *p < 0.05, **p < 0.01, and ***p < 0.001

As shown in Fig. 8a, the ZnO@PDA/Zein nanocomposite film formed a thin film on the implant healing abutment. Figure 8c shows that it was difficult for the pure 0.6 mg/mL ZnO@PDA NPs solution to form a uniform film on the titanium plate, and film required approximately 10 min to form. In contrast, both the 5% pure zein film and the ZnO@PDA/Zein nanocomposite film composed of 50 mg/mL zein and 0.6 mg/mL ZnO@PDA NPs formed a more uniform, pale white film on the titanium plate in approximately 1 min. The formation of a uniform film was attributed to the good dispersibility and film-forming ability of zein [59, 60]. As shown in Fig. 8d, after the scratch test, the critical adhesion force required to fracture the ZnO@PDA/Zein nanocomposite film was approximately 1.22 N, whereas that for the 5% pure zein film was approximately 1.4 N, and there was no significant difference between them (*p* > 0.05). Moreover, the critical adhesion force for the ZnO@PDA NPs was approximately 0.5 N, which was considerably lower than those of the ZnO@PDA/Zein nanocomposite films and the pure zein films. This may be attributed to the formation of hydrogen bonds between zein and PDA [43, 61], which can increase the mechanical strength of the ZnO@PDA/Zein nanocomposite film.

### Antibacterial properties

**Fig. 9:**
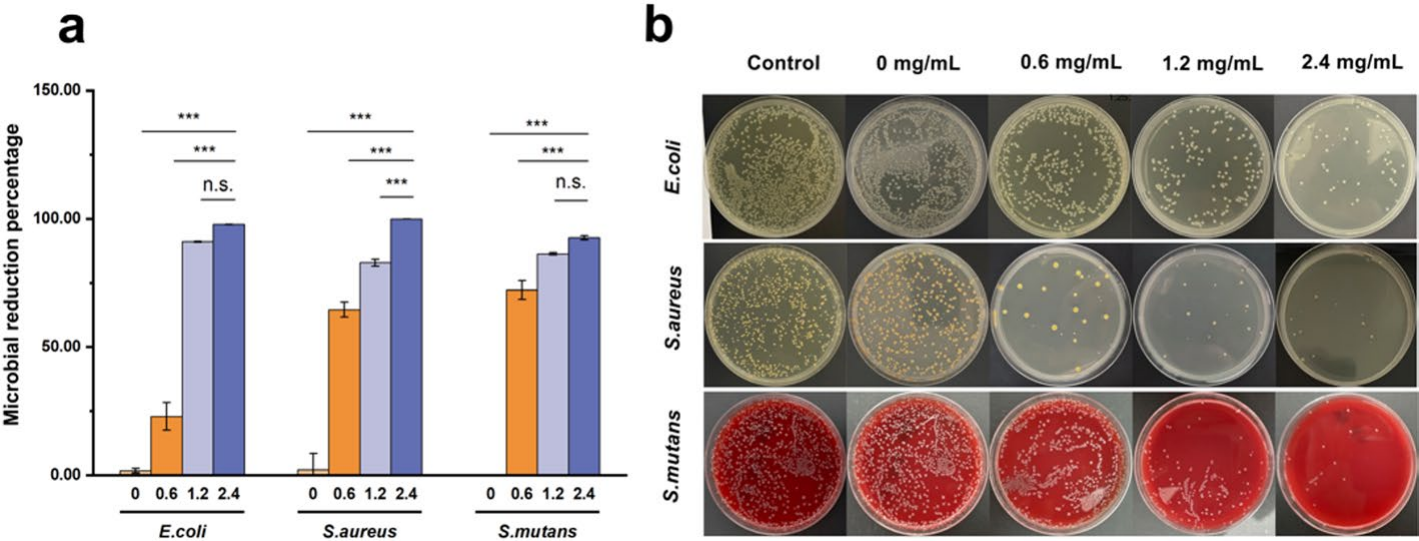
**a** Microbial reduction percentage of ZnO@PDA/Zein nanocomposite films containing different concentrations of ZnO@PDA NPs. **b** Images of *E. coli*, *S. aureus*, and *S. mutans* colony formation after dilution and treatment; *p < 0.05, **p < 0.01, and ***p < 0.001, n.s., not significant

Biocoatings with potent antibacterial activity can effectively eliminate microorganisms at wound sites, thus enhancing the antibacterial functions of therapeutic instruments in the oral cavity. We assessed the in vitro antibacterial properties of zein-based nanocomposite dressings against three representative pathogenic bacteria: *E. coli*, *S. aureus*, and *S. mutans*. As shown in Fig. 9, the pure zein film did not exhibit any antibacterial activity. We found that the ZnO@PDA/Zein nanocomposite film possesses good antibacterial properties and that the antibacterial effects increase with increasing concentrations of ZnO@PDA NPs. When the concentration of ZnO@PDA NPs in the film-forming solution was 0.6 mg/mL, the nanocomposite film had no considerable antibacterial effect on *E. coli*, but the film had antibacterial effects on *S. aureus* and *S. mutans*, with inhibition rates of 96.4% and 76.9%, respectively (Fig. 9a). When the concentration of ZnO@PDA NPs was increased to 1.2 mg/mL, the antibacterial effect against *E. coli* increased, with an inhibition rate of 91.1%, and the inhibition rates against *S. aureus* and *S. mutans* were 98.3% and 88.9%, respectively. As the concentration of ZnO@PDA NPs increased further to 2.4 mg/mL, the inhibition rate of *E. coli* reached 97.2%, whereas the inhibition rates of *S. aureus* and *S. mutans* increased to 99.8% and 93.4%, respectively. The above results indicate that the nanocomposite film has potent antibacterial properties against both Gram-negative and Gram-positive bacteria and that increasing the concentration of ZnO@PDA NPs can enhance the antibacterial effect of the nanocomposite film.

### Biocompatibility

**Fig. 10:**
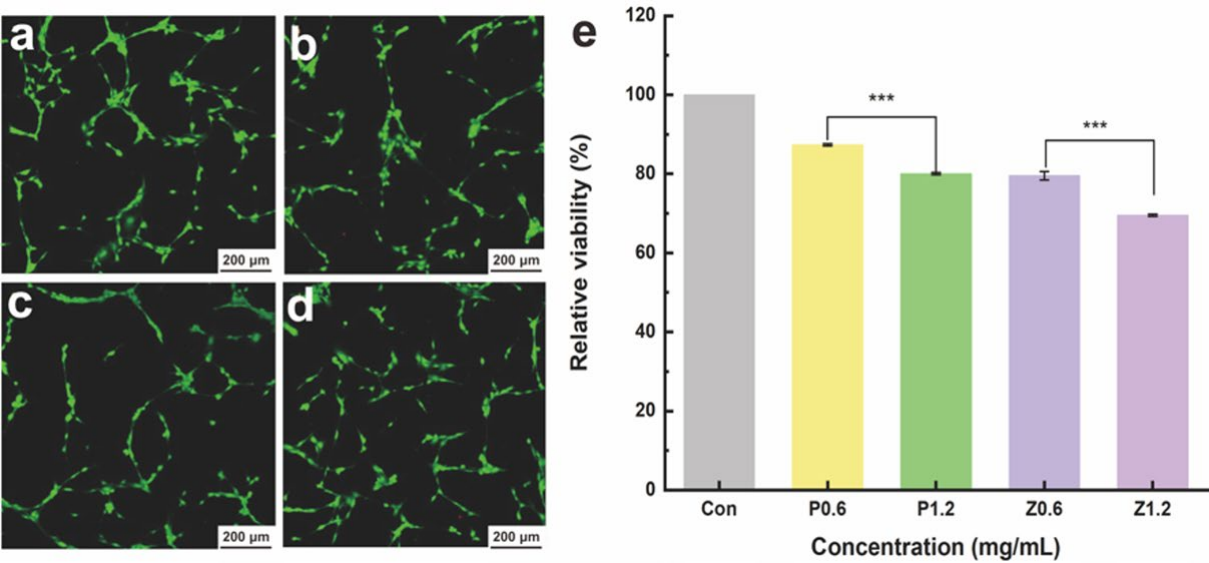
Images of nanocomposite films containing 0.6 mg/mL ZnO@PDA NPs **a**, 0.6 mg/mL ZnO NPs **b**, 1.2 mg/mL ZnO@PDA NPs **c**, and 1.2 mg/mL ZnO NPs **d**. **e** Relative MC3T3-E1 cell survival rates after coculture with the nanocomposite film. Con represents the control group, P0.6 represents the nanocomposite film containing 0.6 mg/mL ZnO@PDA NPs, P1.2 represents the nanocomposite film containing 1.2 mg/mL ZnO@PDA NPs, Z0.6 represents the nanocomposite film containing 0.6 mg/mL ZnO NPs, and Z1.2 represents the nanocomposite film containing 1.2 mg/mL ZnO NPs; *p < 0.05, **p < 0.01, and ***p < 0.001

The biocompatibility of the ZnO@PDA/Zein nanocomposite film was assessed by determining its toxicity to mouse preosteoblasts (MC3T3-E1 cells) using the CCK-8 assay. As depicted in Fig. 10e, MC3T3-E1 cell viability after treatment with the ZnO/Zein film incorporating 0.6 mg/mL ZnO NPs was 78.75%, whereas MC3T3-E1 cell viability decreased considerably to 69.424% after treatment with the ZnO/Zein film with 1.2 mg/mL ZnO NPs following 24 h of incubation. An increase in the concentration of ZnO NPs clearly correlates with an increase in the cytotoxicity of the nanocomposite film [62, 63]. Recent investigations on the biotoxicity of ZnO NPs have focused primarily on the generation of ROS upon catalysis on the surface of ZnO NPs, which, upon cellular internalization, elevates intracellular ROS levels, triggering oxidative stress [64]. Typically, biological systems counteract ROS enzymatically with catalase or superoxide dismutase or via nonenzymatic antioxidants [65, 66]. However, the accumulation of ZnO NPs within an organism can disrupt this equilibrium, resulting in excessive generation of ROS that may induce apoptosis [67]. In this study, we attempted to mitigate the cytotoxicity of ZnO NPs by modifying ZnO NPs with PDA. MC3T3-E1 cell viability after treatment with the nanocomposite film containing 0.6 mg/mL ZnO@PDA was 87.34%, which was considerably greater than that after treatment with the film containing 0.6 mg/mL ZnO/Zein. When the concentration of ZnO@PDA NPs was increased to 1.2 mg/mL, the cell viability reached 78.75%. These findings suggest that modifying ZnO NPs with PDA effectively reduces their cytotoxicity. Confocal laser scanning microscopy revealed that the MC3T3-E1 cells in all the experimental groups maintained a favourable viability morphology and adhered robustly to the nanocomposite film surface, with no evident cell death observed. These findings demonstrate that ZnO@PDA NPs and ZnO NPs at a concentration of 0.6 mg/mL have superior biocompatibility with MC3T3-E1 cells.

## Discussion

In this study, a ZnO@PDA/Zein nanocomposite film was successfully synthesized. Characterization was conducted using TEM, FTIR, and SEM to observe the surface morphology and internal structure of the ZnO@PDA/Zein nanocomposite film and to study its formation time, degradation rate, drug release in vitro, and in vitro antibacterial properties. The TEM images revealed that PDA coated the surface of the ZnO NPs to form a shell-like structure. FTIR spectroscopy also confirmed the successful synthesis of the ZnO@PDA/Zein nanocomposite film. In the degradation study, the nanocomposite film underwent gradual self-degradation within a salivary milieu. After 7 days of incubation, the mass loss rate of the nanocomposite film was approximately 55%. Additionally, the film facilitates controlled release of the encapsulated pharmaceutical agent, with drug release lasting for up to 14 days. The cumulative release of Zn^2+^ over the 14-day period reached approximately 56.33%, indicating that the nanocomposite film was capable of sustained drug delivery.

In the antibacterial activity experiments, the ZnO@PDA/Zein nanocomposite film exhibited propriety against both Gram-positive and Gram-negative bacteria. On the basis of previous research, we hypothesize that the antibacterial activity of the nanocomposite film may involve primarily the direct interaction of released ZnO NPs with pathogenic bacteria, leading to bacterial damage and subsequent death [68]. For example, nanocomposites of various metal oxides can interact directly with negatively charged microbial membranes through electrostatic interactions, complex porosity, or the release of surface ions. This interaction can lead to the disruption of microbial cell walls, allowing the NPs to enter the cytoplasm and disrupt bacterial function by generating superoxide and hydroxyl radicals from membrane proteins [69]. Moreover, the results of our antibacterial experiments showed a positive correlation between the concentration of ZnO NPs within the nanocomposite film and its antibacterial efficacy. This correlation indicates a concentration-dependent mode of antibacterial action. Notably, the nanocomposite film demonstrated enhanced antibacterial activity specifically against *S. aureus* and *S. mutans*, with a less pronounced effect observed against *E. coli*. The antibacterial efficacy of the nanocomposite film is also species dependent. Compared with *S. aureus*, *E. coli* exhibited greater resistance to these films; this may be attributed to the fact that Gram-negative bacteria possess a double membrane bilayer cellular structure, particularly an outer membrane that contains lipopolysaccharides [70]. These structural features enable the outer membrane to serve as a barrier against antibacterial agents. Conversely, the ability of ZnO NPs to directly bind to the porous cell walls of Gram-positive bacteria facilitates their entry into the cells; this results in the leakage of intracellular contents and ultimately leads to cell death [71]. The aggregation of hydrophobic ZnO NPs in aqueous media reduces their ability to interact with microorganisms. PDA modification mitigates this issue by promoting greater contact between ZnO NPs and bacterial cells, thus enhancing antibacterial efficacy [72]. The antibacterial mechanism of ZnO NPs may be related to their particle morphology, as particles with high surface areas have shown greater antibacterial activity [62]. Additionally, the antibacterial activity of ZnO NPs is size dependent, with smaller, higher-concentration NPs being more effective [49]. The ZnO NPs used herein had a mean diameter of approximately 20 nm (Fig. 4). The potential mechanisms of action for metal nanoparticles are as follows: (a) respiratory chain enzyme disruption due to microbial plasma membrane damage, (b) metal ion accumulation within microbial membranes, and (c) inhibition of metabolic processes through electrostatic attraction between metal nanoparticles and microbial cells [72]. Owing to the encapsulation of the ZnO NPs by PDA, the ZnO@PDA/Zein nanocomposite film exhibited good biocompatibility [73]. When the concentration of ZnO@PDA NPs in the film-forming solution was 0.6 mg/mL, the relative viability of the mouse embryo osteoblast precursor cells was 87%, indicating that at this low concentration, the ZnO@PDA/Zein nanocomposite film has modest cytotoxicity and good biocompatibility. As an antioxidant, the PDA coating the surface of the ZnO NPs efficiently reduces the amount of ROS produced on the particle surface [74]. Moreover, evidence suggests that intracellular ROS production is predominantly initiated by the internalization of ZnO NPs or the subsequent release of Zn^2+^ into the cytoplasm [75]. In this study, the ZnO NPs were encapsulated by a PDA shell (Fig. 4f), which limited the release of Zn^2+^ [76]. This controlled release mechanism prevents the burst release of ZnO NPs from the nanocomposite film, resulting in the sustained release of Zn^2+^. This sustained release confers long-term antibacterial activity on the film while maintaining its good biocompatibility [77]. Additionally, PDA prevents agglomeration and promotes uniform dispersion of ZnO NPs in the nanocomposite film, thus enhancing the antibacterial activity of the small-sized NPs [78]. However, when the concentration of ZnO@PDA NPs increased, the cytotoxicity of the nanocomposite film also increased. The ZnO@PDA/Zein nanocomposite film solution can rapidly form a film on the surface of oral treatment devices, which not only reduces the operation duration for doctors but also minimizes contamination and other uncertainties. In saliva, the ZnO@PDA/Zein nanocomposite film is insoluble but decomposes slowly. During decomposition, the film releases Zn^2+^, endowing the oral treatment instruments with antibacterial properties. By adjusting the concentration of the ZnO@PDA NPs, excellent antibacterial effects were achieved. However, the limitation of this study is that while high concentrations of NPs can achieve excellent antibacterial performance, they cannot simultaneously ensure good biocompatibility. Furthermore, investigations into biocompatibility require more prolonged cytotoxicity experiments and observations, which will be the focus of our future research endeavours.

## Conclusion

In summary, we successfully fabricated a ZnO@PDA/Zein nanocomposite film via a solution casting approach. This film offers a facile and cost-effective preparation method coupled with a prolonged sustained-release profile. The nanocomposite film exhibited potent antibacterial activity and favourable biocompatibility through the sustained release of small-sized ZnO@PDA NPs. These properties make this film a promising candidate for surface antibacterial applications on oral therapeutic instruments, particularly implant healing abutments, orthodontic brackets, and cyst obturators. We further envision the application of this film in tissue regeneration.

## Electronic Supplementary Material

Below is the link to the electronic supplementary material.

Supplementary Material 1

## Data Availability

Yes, included in the paper or Supplementary Information (for raw data, not summary data such as means and variances).
